# Analysis of ageing-associated grey matter volume in patients with multiple sclerosis shows excess atrophy in subcortical regions

**DOI:** 10.1016/j.nicl.2016.11.005

**Published:** 2016-11-09

**Authors:** Courtney A Bishop, Rexford D Newbould, Jean SZ Lee, Lesley Honeyfield, Rebecca Quest, Alessandro Colasanti, Rehiana Ali, Miriam Mattoscio, Antonio Cortese, Richard Nicholas, Paul M. Matthews, Paolo A Muraro, Adam D Waldman

**Affiliations:** a Imanova Centre for Imaging Sciences, London, UK; b Division of Brain Sciences, Imperial College London, UK; c Department of Imaging, Imperial College Healthcare NHS Trust, London, UK; d Department of Psychological Medicine, Institute of Psychiatry, Psychology and Neuroscience, King's College London, UK; e Department of Neurology and Psychiatry, Sapienza University of Rome, Italy; f Centre for Clinical Brain Sciences, University of Edinburgh, UK

**Keywords:** Multiple sclerosis, Magnetic resonance imaging, Image analysis, Grey matter, Atrophy

## Abstract

Age of onset in multiple sclerosis (MS) exerts an influence on the course of disease. This study examined whether global and regional brain volumes differed between “younger” and “older” onset MS subjects who were matched for short disease duration, mean 1.9 years and burden as measured by the MS Severity Score and relapses.

21 younger-onset MS subjects (age 30.4 ± 3.2 years) were compared with 17 older-onset (age 48.7 ± 3.3 years) as well as age-matched controls (*n* = 31, 31.9 ± 3.5 years and *n* = 21, 47.3 ± 4.0 years). All subjects underwent 3D volumetric T1 and T2-FLAIR imaging. White matter (WM) and grey matter (GM) lesions were outlined manually. Lesions were filled prior to tissue and structural segmentation to reduce classification errors.

Volume loss versus control was predominantly in the subcortical GM, at > 13% loss. Younger and older-onset MS subjects had similar, strong excess loss in the putamen, thalamus, and nucleus accumbens. No excess loss was detected in the amygdala or pallidum. The hippocampus and caudate showed significant excess loss in the younger group (*p* < 0.001) and a strong trend in the older-onset group.

These results provide a potential imaging correlate of published neuropsychological studies that reported the association of younger age at disease onset with impaired cognitive performance, including decreased working memory.

## Highlights

• Compared brain atrophy within MS cohort differing only by age, and matched controls
• In early MS, subcortical GM atrophy is more than three times that of cortical GM.
• By contrast, WM and cortical GM do not show excess atrophy compared with controls.
• Hippocampal atrophy noted for clinically-matched younger versus older onset MS
• Proposed workflow for atrophy analysis of imaging data in MS

## Graphical abstract

**Figure:**
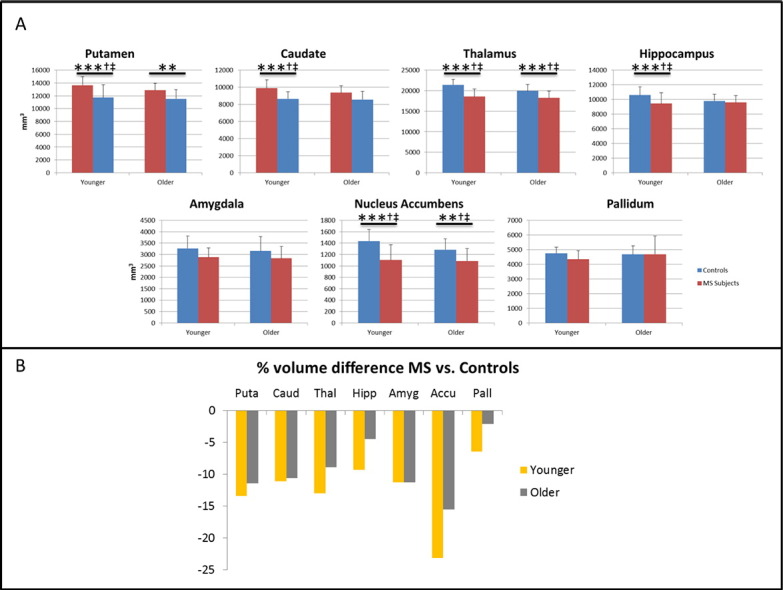

## 1 Introduction

Multiple Sclerosis (MS) is a chronic inflammatory disease of the central nervous system, most commonly presenting in young adults as relapsing remitting (RRMS) or later in life as progressive disease (either primary- or secondary-progressive: PPMS or SPMS, respectively), and associated, especially in the latter, with significant neurodegenerative pathological features. The notion of MS as a disease exclusively affecting the white matter (WM), with multifocal demyelination, is diminishing (Friese et al., 2014, Vigeveno et al., 2012). Indeed, WM lesion load detected by MRI is only weakly correlated with clinical symptoms (Zivadinov and Cox, 2007). There is a growing body of evidence from both pathology and MRI to suggest that grey matter (GM) degeneration is prevalent in MS (Bermel et al., 2003), (Bakshi et al., 2005), (Chard et al., 2004), (Sanfilipo et al., 2006) and may be a stronger predictor of clinical decline than WM measures (Fisniku et al., 2008, Pirko et al., 2007). Information pertaining to the relative atrophy rate of cortical and subcortical GM regions in early MS, and their role in the disease course, remains a topic of study (Bergsland et al., 2012).

Studies of GM atrophy in MS can be confounded by segmentation errors arising from the MS lesions, which result in misclassification of WM regions as GM, and vice versa. One way to circumvent this issue is to mask the lesion areas after global tissue segmentation, but the segmentation error could potentially extend beyond the locality of the lesion itself. Thus, lesion filling prior to segmentation of the anatomical MRI data has been proposed (Chard et al., 2010), and has shown superior segmentation results over retrospective lesion masking (Battaglini et al., 2012). However, lesion filling is not yet universally adopted in image analysis procedures.

The age of onset of MS is relatively varied and it is yet unclear what role GM changes play in this. An older age of RRMS onset is associated with an increased risk of conversion to the more disabling SPMS, independent of disease duration and early relapse frequency (Scalfari et al., 2011). Transformation from RRMS to SPMS may therefore be in part a consequence of ageing itself, rather than solely due to primary, disease-specific pathology. In other work, myelin integrity (as measured by magnetization transfer MRI) has been shown to be independently affected by age in early MS (Newbould et al., 2014), so GM may be similarly affected.

The objectives of this study are therefore three-fold: (i) to establish whether cortical or subcortical GM atrophy dominates in early MS; (ii) to investigate whether the degree of GM atrophy differs between younger and older-onset MS patients of matched clinical status – reflecting the effect of age on CNS damage and repair, independent of disease duration; and (iii) to propose an image analysis workflow for reproducible and standardized quantification of the required imaging endpoints for such analysis.

We recruited two age groups of MS patients with short disease duration from within a well-characterised research cohort. The only factor of significant demographic difference between the two MS groups was age. Comparison of these with ‘younger’ and ‘older’ groups of age-matched healthy controls enabled cross-sectional analysis of GM (and WM) atrophy in each age group.

## 2 Methods

### 2.1 Subjects

A total of 38 MS patients and 52 age-matched controls were involved in this study (Table 1). Subjects gave written informed consent, and the studies had ethical approval from the National Research Ethics Service.

### 2.2 MRI acquisition

All subjects were imaged on a 3T Verio clinical MR system (Siemens Healthcare, Erlangen, Germany; VB17), using a 12-channel phased-array head coil. A T1-weighted 3D MPRAGE volume acquisition was based on the ADNI-GO recommended parameters (Jack et al., 2008) but with 1 mm^3^ isotropic resolution and parallel imaging (PI) factor of 2. A T2-weighted fluid-attenuated inversion recovery (T2-FLAIR) volume was acquired with 1 mm^3^ isotropic resolution using a 3D T2w variable-refocusing angle turbo spin echo readout (Mugler and Brookeman, 2003), with 160 sagittal sections captured in a single 3D slab with the following parameters: echo time (TE) 395 ms, repetition time (TR) 5 s, inversion time (TI) 1800 ms, 250 × 250 mm field-of-view, and a PI factor of 2.

### 2.3 Data analysis

**Fig. 1:**
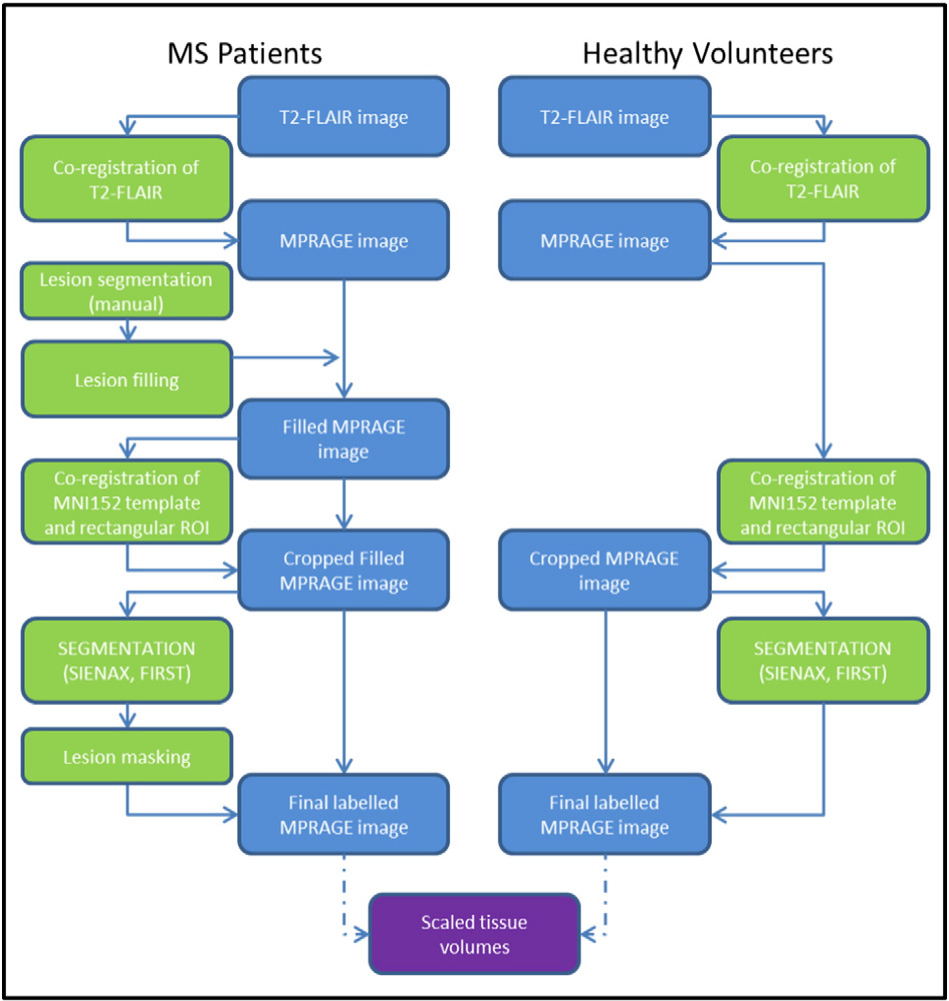
Image analysis workflow. The image analysis workflow for extracting measures of scaled tissue volumes from the input T1-weighted MPRAGE scans of MS patients (left) and healthy controls (right). Blue boxes signify 3D image volumes, whilst green boxes signify processing steps. The final numerical outputs are colored in purple.

Images were processed following the analysis workflow depicted in Fig. 1. Each subject's T2-FLAIR volume was co-registered to their MPRAGE using the rigid-body transformation of FLIRT (Jenkinson et al., 2002). To improve the subsequent tissue segmentations (Popescu et al., 2012), the MNI152 template was aligned using the affine registration of FLIRT (Jenkinson et al., 2002) to the same MPRAGE. This transform was then applied to a rectangular mask covering the MNI152 template, and the transformed mask applied to the MPRAGE to remove excess neck that can corrupt brain extraction tools (Popescu et al., 2012). After masking, the anatomical scans were segmented using additional FSL tools: (i) SIENAX (Smith et al., 2002) for scaled tissue volumes of WM, GM, and cortical GM, normalized for subject head size; and (ii) FIRST (Patenaude et al., 2011) for subcortical grey matter volumes of the putamen, caudate, thalamus, hippocampus, amygdala, accumbens, and pallidum. Subcortical GM structures were multiplied by the SIENAX volume-correction factor to normalize the volumes across subjects. For the MS patients, additional processing steps were performed. WM and GM lesions were filled (Battaglini et al., 2012) using the manually defined lesion masks before the tissue segmentations. After segmentation, correct assignment of WM and GM lesions to WM and GM respectively was checked by masking the partial volume estimates inside the lesion masks. Finally, the recommended brain extraction tool (BET) parameters (of B and f = 0.1) for bias field estimation with SIENAX in MS subjects (Popescu et al., 2012) were used.

**Fig. 2:**
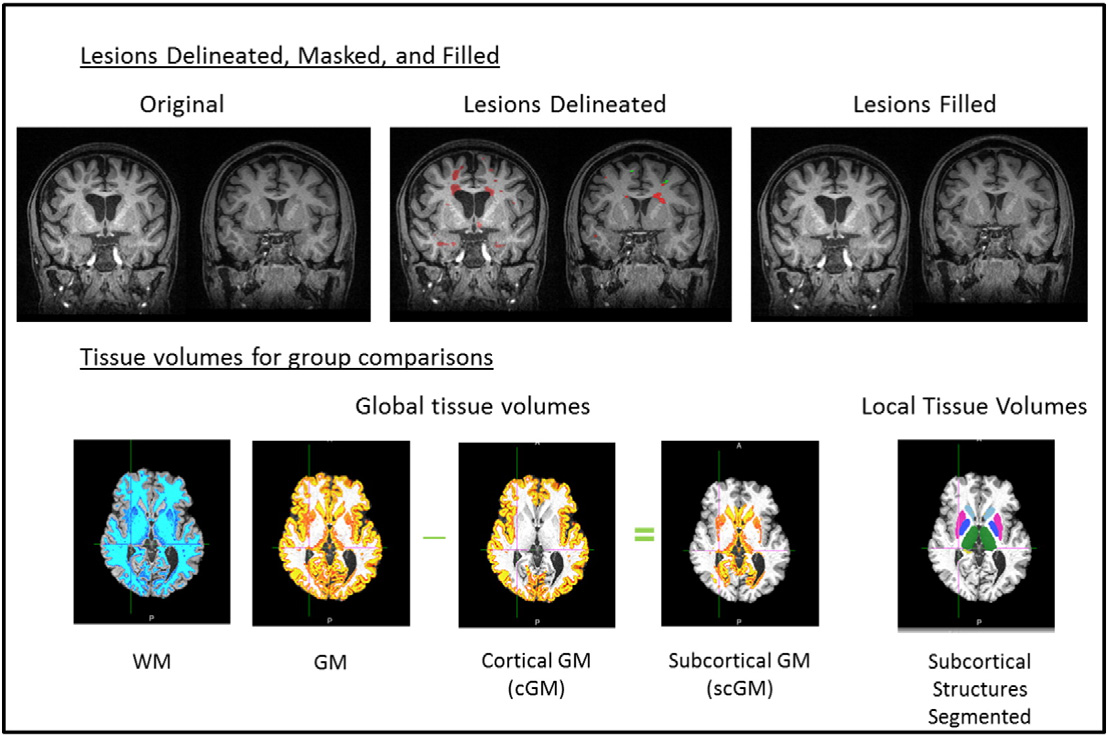
Sample tissue segmentations after lesion masking. Top: Manually delineated lesions, shown for two subjects (red = WM lesions, green = GM lesions) are filled before segmentation to reduce classification errors. Bottom: Tissue volumes as defined with SIENAX for global scaled volumes, and local (subcortical) tissue volumes as defined with FIRST.

Lesion segmentation was performed using a semi-automated intensity-based thresholding technique with manual correction (Jim Version 6.0, Xinapse) by a trained observer and corroborated by a second experienced neuroradiologist, both blinded to subject age and clinical status. GM lesions were segmented from T2-weighted FLAIR images and confirmed on the T1-weighted MPRAGE images. FLAIR images were used for WM lesion definition, due to high lesion conspicuity and detectability on these scans. Fig. 2, top row, shows the segmentation results of the GM and WM lesions in one MS patient.

The scaled SIENAX output volumes (in units of mm^3^) of GM, WM, and cortical GM (cGM) summed across each hemisphere are termed the ‘global’ tissue volumes in this study and were used for comparison of global tissue volumes across the different groups. We also computed a tissue volume for the subcortical and nonperipheral GM (scGM), by subtraction of the scaled cGM from the scaled GM volume, giving a fourth global tissue volume for group comparison (Fig. 2: bottom row). It should be noted that this scGM volume is not an accurate segmentation of subcortical deep grey matter structure volumes, but provides a first-pass indication of the subcortical tissue volume differences between the groups before formal segmentation of individual subcortical grey matter structure volumes (Fig. 2: bottom right). For example, the scGM includes the allocortex structures such as the hippocampus. The scaled FIRST output volumes for the defined individual deep grey matter regions (also in units of mm^3^) were compared across groups, to investigate ‘local’ (subcortical) tissue volume changes.

Younger and older groups were combined to investigate tissue volume differences between controls and MS patients. Then, age-group related differences were explored. Differences for age were assessed using a univariate analysis of variance (ANOVA) with four groups. For the two MS groups, differences for the Expanded Disability Status Scale (EDSS), MS Severity Score (MSSS) (Roxburgh et al., 2005), and disease duration were assessed using a Student's *t*-test, whilst gender differences were explored with a Chi-squared test. For volumetric analysis, ANCOVA was used with either two or four groups as the fixed, between-subjects factor, and gender as a covariate throughout. In the two-group analysis where younger and older groups were combined, age was also used as a covariate. All statistical tests were performed in SPSS v20.0 (IBM Corporation, Armonk NY). All *p*-values are reported without correction for multiple comparisons. Comparisons that survive Bonferroni correction for the multiple comparisons amongst groups or regions with a threshold of *p* < 0.05 are marked in the figures and tables.

## 3 Results

### 3.1 Subjects

The demographics of the younger and older MS patients and controls are summarised in Table 1. The mean age at the time of the MRI scan was 31.9 ± 3.5 years (mean ± SD) in the younger control group (*n* = 31, 18M/13F), 30.4 ± 3.2 years in the younger MS group (*n* = 21, 2M/19F), 47.3 ± 4.0 years in the older control group (*n* = 21, 12M/9F) and 48.7 ± 3.3 years in the older MS group (*n* = 17, 5M/12F). According to the revised McDonald's criteria, a diagnosis of RRMS was made for all of the younger MS patients and 15 (out of 17) of the older MS patients. Gender differences were found between the two younger groups and also between the younger MS and the older controls. EDSS was greater in the older group, though the MSSS did not differ between MS groups. The number of recent relapses (2.0 ± 0.8 versus 1.8 ± 0.8) also did not differ between the MS groups.

### 3.2 Global GM and subcortical GM atrophy matched in each age group

**Fig. 3:**
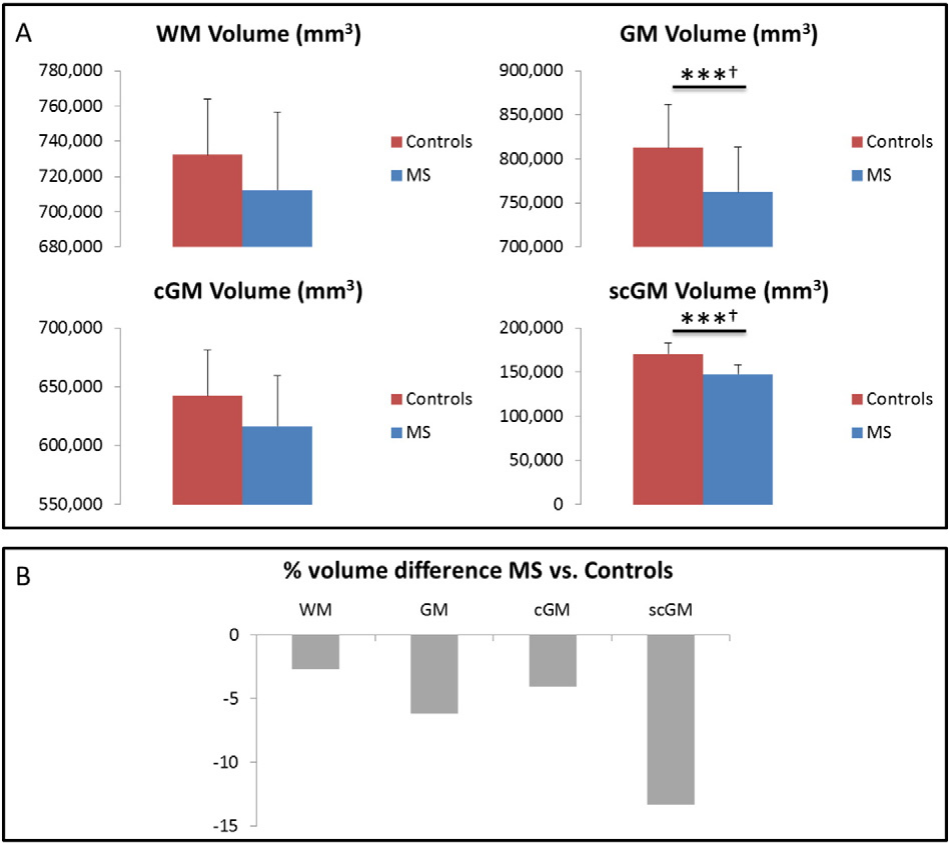
Global tissue volumes: two groups. A: absolute values of the global tissue volumes for all of the controls and the MS patients (i.e., without separation by age group). B: percentage volume decrease of MS global tissue volumes (expressed as a percentage of the control group mean tissue volume). Error bars are standard deviations. (***) *p* ≤ 0.001, uncorrected. (†)survives Bonferroni correction for comparisons amongst groups.

Fig. 3a shows the scaled global tissue volumes (WM, GM, cGM, scGM) for all controls and MS patients without stratifying by age: there is a significant decrease in global GM and subcortical GM (scGM) volume, but not in WM or cortical GM (cGM). The percentage differences in mean global tissue volumes for the MS group relative to the mean volumes for the control group are given in Fig. 3b. Interestingly, the WM volume difference (− 2.74%) is less than half that of the GM (− 6.19%), and the decrease in mean scGM volume (− 13.34%) is more than three times greater than that of the cGM (at − 4.08%) in this cohort.

**Fig. 4:**
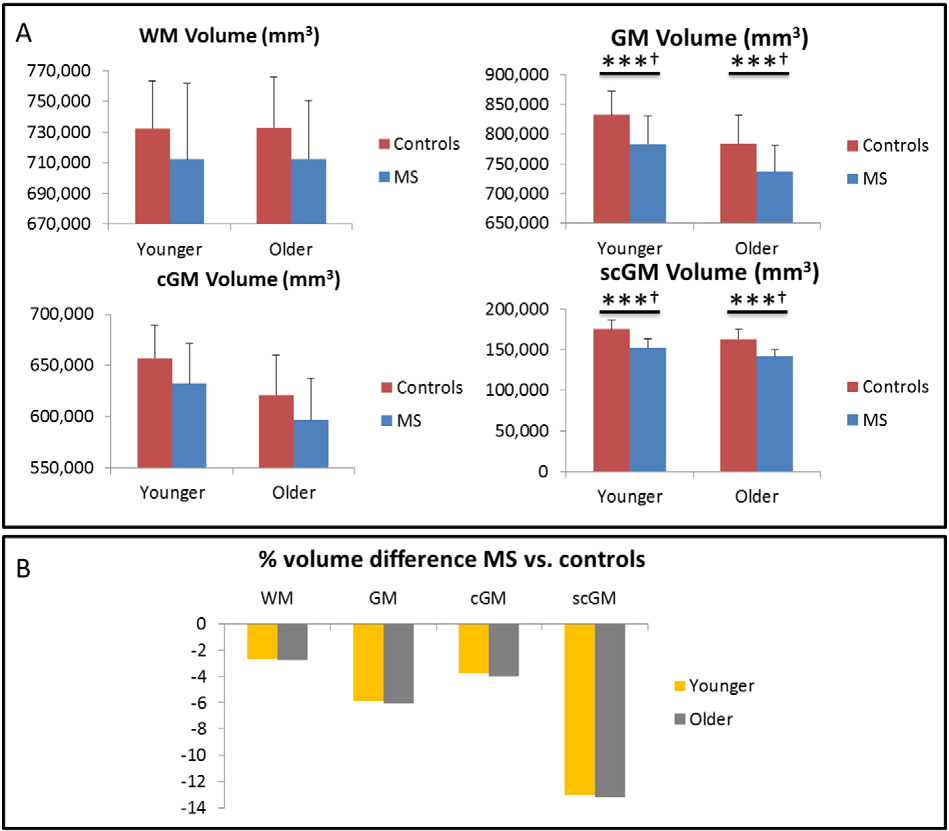
Global tissue volumes: four groups. A: global tissue volumes for both age groups of the controls and the MS patients. B: volume difference for the MS global tissue volumes, expressed as a percentage change from the corresponding control group mean tissue volume. Error bars are standard deviations. (***) *p* ≤ 0.001, uncorrected. (†) survives Bonferroni correction.

Investigating differences within each age group, the plots in Fig. 4a and the data in the top third of Table 2 reveal significant GM and scGM volume differences between age-matched MS patients and controls (younger: *p* < 0.001 for both GM and scGM; older: *p* = 0.001 for GM, *p* < 0.001 for scGM), whilst there is no significant difference between age-matched patients and controls for the WM and the cGM global tissue volumes. Additionally, Fig. 4b reveals that the percentage decreases in mean global tissue volumes relative to controls are comparable for the younger and the older MS group (WM: − 2.72% and − 2.78%; GM: − 5.86% and − 6.08%; cGM: − 3.73% and − 3.99%; scGM: − 13.01% and − 13.18%, respectively). Once again, the greatest atrophy is found in the scGM global tissue volume, explored further by the evaluation of the local (subcortical) tissue volumes below.

### 3.3 Local (subcortical) GM atrophy matched in each age group with notable exceptions

**Fig. 5:**
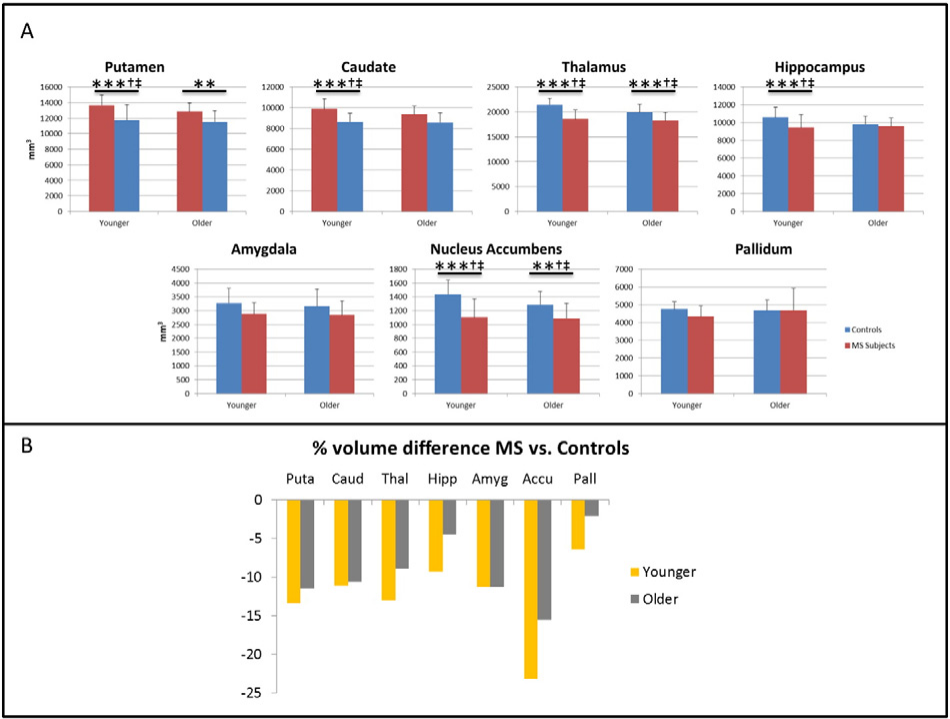
Local tissue volume differences for MS patients. Local (subcortical) GM volume differences for the younger and older MS patients, (A) raw values in mm^3^ and (B) expressed as a percentage change from the corresponding control group mean tissue volumes. (**) *p* < 0.01, (***) *p* < 0.001, ANCOVA of normalized volumes versus age-matched control group. *p*-Values uncorrected; (^†^)survives Bonferroni correction for comparisons amongst groups or (^‡^) regions of interest.

Local (subcortical) GM tissue volumes and ANCOVA results for comparisons across the four groups are presented in the lower two-thirds of Table 2, with atrophy measures (expressed as the percentage change in mean volume relative to the corresponding control group) shown in Fig. 5. Both the younger and older MS patients had significantly reduced local GM volume compared with the age-matched controls in the region of the putamen (*p* < 0.001 for both), thalamus (*p* < = 0.001 for both) and nucleus accumbens (*p* < 0.001 for younger, *p* = 0.005 for older MS patients). In two regions – the caudate and the hippocampus - there was significantly reduced GM volume for only the younger MS patients (*p* < 0.001 for both). The volumes of the amygdala and the pallidum were not significantly different between the MS patients and their age-matched control group (Table 2).

## 4 Discussion

This study investigated both global and local tissue volume differences in younger and older-onset MS patients from a well-characterised cohort with age-matched controls. The two MS groups were selected for short clinical disease duration and matched in disease severity measured by MSSS and recent relapses. For the two-group comparison, there was much less WM atrophy than GM atrophy in MS patients, and the percentage volume changes for cerebral GM and WM reported herein (− 6.19% and − 2.74%, respectively) are comparable to values reported elsewhere for similar-age, but much longer disease duration (mean 11.3 years) MS patients (Ramasamy et al., 2009). Whilst studies have previously identified subcortical GM atrophy in MS patients (Cifelli et al., 2002), (Chard et al., 2004), (Bakshi et al., 2005), (Prinster et al., 2006), (Ramasamy et al., 2009), (Audoin et al., 2010), we believe that this is the first study to directly compare the relative volume loss in younger and older-onset MS of matched early clinical status.

We found that the scGM degeneration dominates over that of the periphery. The definition of scGM included deep grey matter structures as well as non-peripheral (e.g. not neocortex) GM, however similar volume differences were found scGM and in the defined GM structures. There is a body of evidence to suggest preferential degeneration of the subcortical, rather than the cortical, GM tissue in MS patients. The timecourse of this degeneration is less well explored. Bergsland et al. (2012) found that patients with early RRMS had significantly lower subcortical deep GM volumes, but not lower cortical volumes, compared to Clinically Isolated Syndrome (CIS) patients. Findings of significant regional atrophy in the thalamus and putamen are similarly supported by Ramasamy et al. (2009), whilst their observation of visible hippocampal atrophy only in progressive disease stages is contrary to the significant hippocampal atrophy in our younger cohort of early-onset RRMS patients (disease duration of 2.29 ± 1.6 years). Our findings however are consistent with a number of other studies of GM (Roosendaal et al., 2011) and hippocampal (Sicotte et al., 2008) atrophy in RRMS and SPMS.

The novel feature and main focus of this study was to explore brain volume differences in the clinically-matched older and younger-onset MS groups; the rationale being that significant differences might offer insight into the variable age of disease onset and enable inferences on the early-phase trajectory of CNS atrophy in these different age groups. Furthermore, whilst this is not a longitudinal study, comparison of the tissue volume differences in the younger and older MS patients against their corresponding age-matched controls gives a measure of volume loss separate from ‘normal’ age-related decline.

Longitudinal studies in RRMS (as well as PPMS and SPMS), have suggested that the trajectory of CNS atrophy is initially rapid then tails off later in the disease course (Zivadinov and Bakshi, 2004). However, it remains unknown whether the initial rate of CNS atrophy differs between the younger- and older-onset MS patients during the first few years of disease and we sought to ascertain any differences detectable by cross-sectional analysis within 5 years from clinical onset.

We were unable to differentiate volume decreases in the younger and older onset subjects at the global tissue structure level (WM, GM, cGM and scGM). The percentage decrease in mean global tissue volumes for the younger and older MS patients relative to age-matched controls was similar. However, at a local/regional level, findings suggest that some structures, in particular the hippocampus, have unique patterns of involvement. The notable loss in the young-onset and relative preservation in the older-onset MS patients may be indicative of early and marked loss of neurons in hippocampal substructures, as seen in post-mortem studies (Papadopoulos et al., 2009). Chronic GM neuroinflammation as confirmed by in-vivo PET imaging (Colasanti et al., 2015) has been observed in the hippocampus of MS patients. Alternatively, there may be more hippocampal atrophy in younger-onset MS patients from the selective vulnerability of neuronal subpopulations or growth factor dysregulations (Geurts and Barkhof, 2008), or disease heterogeneity resulting in an earlier disease onset. A number of studies have reported worse cognitive dysfunction in early onset MS, most clearly demonstrated in pediatric and juvenile MS (Cardoso et al., 2015, Krysko and O'Connor, 2016). In adult onset MS, left hippocampal atrophy was shown to be correlated with verbal memory performance (Sacco et al., 2015). Our observations of low hippocampal volume may provide an imaging correlate of the more marked cognitive impairment previously demonstrated in younger onset MS (Hosseini et al., 2014). Demonstration of this putative association is, however, limited by the lack of neuropsychometric data on the current cohort.

In addition to the above findings, we have demonstrated an image analysis workflow for analysis of global and local tissue volumes in larger MS studies. A number of fully-automated solutions are being explored (Jain et al., 2015); however in this workflow we opted for manual delineation of both white and grey matter lesions. Lesions were then filled to allow automated segmentation of tissues to produce accurate volume measures of tissues and structures independent of lesion burden. We hope that this workflow will be adopted to facilitate direct comparison of future measures with those reported herein.

Our study does have limitations: (1) cross-sectional design; (2) small sample size; (3) the protocol did not include double inversion recovery sequences, useful to detect hippocampal focal lesions; and (4) neuropsychological assessments were not performed.

However, our results confirm previous studies demonstrating GM atrophy in MS and considerably extend them with the novel detection of excess volume decreases in subcortical regions, particularly the hippocampus, that play a central role in memory processing.

Further longitudinal imaging studies incorporating cognitive testing are warranted to understand in more depth the development of regional GM atrophy and any regional differences in patients with demyelinating disease (including radiologically- and clinically isolated syndromes; and MS), and its relationship with age and cognitive function.

## Funding sources

The Patient Research Cohort “Rapidly Evolving MS” was funded by the UK Medical Research Council (ref. no. G0800679) and supported by the NIHR CRF and BRC at Imperial College Healthcare NHS Trust. The views expressed are those of the authors and not necessarily those of the MRC, the NHS, the NIHR or the Department of Health.

## Figures and Tables

**Table 1:** Demographics: four groups. Table of demographics for the four subject groups (both controls and MS patients, young and old) and results of group comparisons. Values reported as mean (SD). NA = not applicable.

	Younger Control	Younger MS	Older Control	Older MS	*p* (yC/yMS)	*p* (yC/oC)	*p* (yC/oMS)	*p* (yMS/oC)	*p* (yMS/oMS)	*p* (oC/oMS)
N	31	21	21	17						
Age (years)	31.9 (3.5)	30.4 (3.2)	47.3 (4.0)	48.7 (3.3)	0.86	< 0.001 expected	< 0.001 expected	< 0.001 expected	< 0.001 expected	1.00
Gender (M/F)	18/13	2/19	12/9	5/12	< 0.001	1.00	0.75	0.003	0.21	0.11
D.D. (years)	NA	2.3 (1.6)	NA	2.4 (1.2)	NA	NA	NA	NA	0.89	NA
EDSS	NA	3.0 (1.2)	NA	4.1 (1.3)	NA	NA	NA	NA	0.008	NA
MSSS	NA	5.5 (1.6)	NA	6.3 (1.7)	NA	NA	NA	NA	0.13	NA
Relapses (#)	NA	2.0 (0.8)	NA	1.8 (0.8)	NA	NA	NA	NA	0.50	NA
		All RRMS		15 RRMS 2 SPMS						

**Table 2:** Tissue volumes and comparisons: four groups. Tissue volumes and Analysis of Covariance (ANCOVA) results for the four subject groups (both MS patients and controls, young and old). Values reported as mean (SD). All *p*-values are uncorrected for multiple comparisons. Daggers denote values that survive Bonferroni correction for comparison with multiple groups (^†^) or regions of interest (^‡^).

	Younger control	Younger MS	Older control	Older MS	*p* (yC/yMS)	*p* (yC/oC)	*p* (yC/oMS)	*p* (yMS/oC)	*p* (yMS/oMS)	*p* (oC/oMS)
*Global level*										
White	732,105.8 (31,001.2)	712,226.7 (49,240.6)	732,776.1 (33,148.3)	712,422.9 (38,048.7)	0.16	0.95	0.14	0.17	0.92	0.15
Grey	831,717.9 (39,916.0)	782,947.7 (47,128.5)	784,524.5 (47,578.4)	736,858.7 (44,119.2)	< 0.001^†‡^	< 0.001^†‡^	< 0.001^†‡^	0.72	0.003^†‡^	0.001^†‡^
cGM	656,787.4 (32,131.5)	632,263.7 (39,229.3)	621,226.4 (38,781.6)	596,443.2 (40,896.4)	0.011^†‡^	0.001^†‡^	< 0.001^†‡^	0.60	0.007^†‡^	0.028
scGM	174,930.6 (11,659.8)	152,175.8 (10,721.2)	163,298.2 (11,037.0)	141,774.5 (8627.0)	< 0.001^†‡^	< 0.001^†‡^	< 0.001^†‡^	0.006^†‡^	0.003^†‡^	< 0.001^†‡^

*Regional level*										
Puta	13,658.9 (1312.6)	11,727.6 (1971.7)	12,850.3 (1106.1)	11,530.9 (1437.0)	< 0.001^†‡^	0.06	< 0.001^†‡^	0.25	0.68	0.009
Caud	9888.8 (965.2)	8647.7 (842.7)	9352.6 (830.6)	8556.9 (973.3)	< 0.001^†‡^	0.04	< 0.001^†‡^	0.03	0.75	0.012
Thal	21,405.1 (1292.5)	18,573.1 (1873.0)	19,924.1 (1609.5)	18,246.5 (1629.5)	< 0.001^†‡^	0.001^†‡^	< 0.001^†‡^	0.002^†‡^	0.69	0.001^†‡^
Hipp	10,598.2 (1131.5)	9452.6 (1438.6)	9807.3 (926.0)	9579.6 (953.0)	< 0.001^†‡^	0.014	0.001^†‡^	0.11	0.55	0.33
Amyg	3271.4 (543.1)	2878.9 (411.8)	3156.5 (619.9)	2835.4 (525.1)	0.026	0.45	0.012	0.15	0.77	0.09
Accu	1439.3 (199.7)	1104.1 (266.9)	1284.6 (191.2)	1088.2 (215.7)	< 0.001^†‡^	0.014	< 0.001^†‡^	0.005^†‡^	0.94	0.005^†‡^
Pall	4740.7 (430.7)	4343.2 (584.7)	4679.7 (587.4)	4686.0 (1237.3)	0.03	0.76	0.65	0.08	0.11	0.87
